# LLM-powered prostate cancer staging from PSMA-PET/CT reports using PROMISE v2

**DOI:** 10.1007/s00259-026-07847-w

**Published:** 2026-03-21

**Authors:** Daniel Spitzl, Markus Mergen, Lukas Endroes, Christopher Spaeth, Rupert Trager, Rickmer F. Braren, Matthias Eiber, Lisa Steinhelfer

**Affiliations:** 1 Department of Diagnostic and Interventional Radiology, School of Medicine, TUM University Hospital, Technical University of Munich, Ismaningerstr. 22, Munich, 81675 Germany; 2 Department of Diagnostic and Interventional Radiology and Nuclear Medicine, University Medical Center Hamburg-Eppendorf, Hamburg, Germany; 3 Department of Nuclear Medicine, School of Medicine and Health, TUM University Hospital, Technical University Munich, Munich, Germany; 4 Department of Diagnostic and Interventional Neuroradiology, Klinikum rechts der Isar, School of Medicine and Health, Technical University Munich, Munich, Germany; 5 German Cancer Consortium (DKTK), partner-site Munich, DKFZ and Klinikum rechts der Isar, Munich, Germany; 6 Bavarian Cancer Research Center (BZKF), Munich, Germany

**Keywords:** LLM, PROMISE v2, PSMA-PET/CT, Prostate cancer, TNM staging

## Abstract

Accurate staging of prostate cancer is essential for guiding therapy and predicting outcomes. Prostate-specific membrane antigen (PSMA) PET/CT has become an established modality for disease assessment, and the PROMISE v2 framework provides standardized criteria for molecular imaging–based TNM (miTNM, molecular imaging TNM) classification. This study investigates the potential of large language models (LLMs) to automatically extract PROMISE v2 staging information from PSMA PET/CT reports. We retrospectively analyzed 1,696 reports from first-diagnosis prostate cancer patients using the open-source Meta-Llama-3.1-8B-Instruct (in the Q8_0 GGUF quantization) model, deployed securely within institutional infrastructure. Four prompting strategies were systematically compared: Zero-shot, advanced Zero-shot, few-shot, and chain-of-thought (CoT). Performance was evaluated using accuracy, precision, recall, and micro- and macro-averaged F1 scores. Advanced Zero-shot prompting achieved the highest performance for local staging (micro-F1 = 0.65), while CoT prompting was superior for nodal (micro-F1 = 0.79) and metastatic staging (micro-F1 = 0.84). Error analyses revealed that Zero-shot approaches tended to collapse predictions into central categories, whereas advanced Zero-shot and CoT prompting yielded more balanced and stage-specific outputs. These findings demonstrate that LLMs can reliably map narrative PET/CT reports onto structured PROMISE v2 staging categories, with prompting strategy strongly influencing performance across staging dimensions. Our results highlight the feasibility of LLM-based workflows for standardizing prostate cancer staging, enabling reproducibility, and supporting large-scale outcome analyses.

**Supplementary Information:**

The online version contains supplementary material available at 10.1007/s00259-026-07847-w.

## Introduction

Prostate cancer is the second most frequently diagnosed malignancy in men and remains a major contributor to cancer-related mortality worldwide [1, 2]. Given its global burden, accurate disease characterization is essential for effective clinical management, as therapeutic strategies and prognosis vary substantially between localized, locally advanced, and metastatic stages [3, 4]. In this context, Prostate-specific membrane antigen (PSMA) targeted positron emission tomography (PET) has emerged as a pivotal imaging modality for staging and restaging of patients with prostate cancer [5–8]. To facilitate standardized reporting and to improve objectivity and accuracy in image interpretation for PSMA-PET, the prostate cancer molecular imaging standardized evaluation (PROMISE) framework was published in 2018 and updated in 2023 and defines criteria for PSMA-PET standardised assessment [9, 10]. PROMISE was developed to categorise clinically relevant disease stages by mapping whole-body PSMA-PET findings into molecular imaging TNM stages, including quantifiable tumour volume and target expression (SUV_mean_) [9]. 

In parallel, with advances in artificial intelligence, large language models (LLMs) have emerged as valuable tools for analyzing medical texts, including radiology reports, and have shown considerable potential in clinical applications [11–13]. Their ability to process complex medical texts raises the prospect of leveraging them for oncologic classification tasks. While LLMs have already been applied for PET/CT staging in other tumor entities, such as breast cancer, their use in prostate cancer remains largely unexplored [14–16].

Building on these developments, LLMs may facilitate extraction of PROMISE classifications from PSMA-PET/CT reports, thereby standardizing TNM staging, improving reproducibility, and supporting clinical decision-making. Since cloud-based systems (e.g., ChatGPT, Gemini) cannot be employed for sensitive patient data, we locally implemented an open-source LLM to extract and structure information from PSMA-PET/CT reports. This study evaluates the potential of LLMs for prostate cancer staging, with a focus on their capacity to reliably interpret and apply PROMISE v2 criteria.

## Materials and methods

### Patient selection and study design

All analyses were conducted under ethics approval (reference 87/18S) from institutional review board of the Technical University of Munich, with all methods carried out in compliance with relevant guidelines and regulations. Because of the retrospective nature of this study, the requirement for written informed consent was waived by the institutional review board.

We reviewed 1,696 patients who underwent PSMA-PET/CT examinations at our institution between June 2022 and June 2025. All reports, written in German, were authored by certified nuclear medicine specialists. We reviewed a total of 5,360 entries from our institutional database. The final cohort was selected through a two-stage filtering process: First, patients without a primary tumor (T0; *n* = 2,565), recurrences (Tr; *n* = 936), and technical duplicates (*n* = 44) were excluded. From the remaining 1,815 cases, patients with T1 stage (*n* = 12) and reports containing ambiguous findings that precluded definitive staging (*n* = 107) were removed. Consequently, a total of 1,696 patients were included in the final analysis. The selection process, including inclusion and exclusion criteria, is detailed in the study flowchart ( Suppl. Figure 1).

### Reference standard (ground truth)

To establish the reference standard, all 1,696 reports were manually annotated by two readers (first a nuclear medicine resident and for validation a board-certified nuclear medicine specialist with > 5 years of experience in PSMA-PET/CT). The readers assigned T, N, and M categories according to the PROMISE v2 framework based on the textual information in the clinical reports. In cases of discrepancy a final label was reached by consensus.

### LLM framework and prompting strategies

The LLM utilized throughout the study was Meta-Llama-3.1-8B-Instruct (in the Q8_0 GGUF quantization). This model was selected for its enhanced multilingual capabilities and its ability to be deployed securely within institutional infrastructure, ensuring compliance with data privacy and security standards (e.g., GDPR). To ensure data privacy and local processing, all experiments were conducted on a dedicated virtual machine (VM). The hardware configuration consisted of an AMD EPYC processor (16 cores) and 128 GiB of RAM. Model inference was accelerated using a virtualized NVIDIA H100 GPU (Hopper architecture) with 24 GB of vRAM. The software environment was based on CUDA 13.0 and NVIDIA driver version 580.82.07. No model fine-tuning was performed to maintain the generalizability of the base model. Instead, four prompting strategies were systematically tested to guide the model’s staging assessment:

- Zero-shot prompting: The task was executed using a Zero-shot prompting configuration, in which the model received only a natural language description of the task and the target classification criteria. No task-specific examples or expert-annotated demonstrations were provided within the prompt context. This approach was chosen to evaluate the model’s intrinsic Zero-shot generalization capabilities and its ability to map textual findings to categories based solely on the semantic understanding acquired during its pre-training phase.
- adv. Zero-shot prompting: To evaluate the model’s ability to apply complex medical logic, we utilized an advanced Zero-shot configuration. The prompt was enriched with explicit domain-specific constraints and the full taxonomy of staging criteria. This allowed the model to perform deductive reasoning based on the provided framework, ensuring that the classification was grounded in established clinical guidelines rather than relying solely on latent pre-trained associations.
- Chain-of-thought + self-consistency prompting: We utilized a Chain-of-Thought (CoT) approach integrated with a self-consistency framework. For each input, the model was prompted to articulate its reasoning step-by-step. To ensure logical reliability, we generated parallel reasoning trajectories for each query. The final classification was determined by selecting the most frequent result among these trajectories, prioritizing the consensus of the model’s independent ‘thought’ paths over any single iteration.
- Few-shot prompting: The model was provided with five manually selected, expert-annotated clinical reports as examples within the prompt to illustrate the desired mapping of textual findings to PROMISE v2 categories.

Prompts can be found in Suppl. Table 1.

Performance was assessed using accuracy, precision, recall, micro and macro-averaged F1 scores across the staging categories. The F1 score was calculated as the harmonic mean of precision (also known as positive predictive value) and recall (also known as sensitivity). The micro scores were computed by aggregating the true-positive, false-negative, and false-positive findings across all classes. The macro scores were computed by calculating the scores for each class individually and then averaging them, giving equal weight to each class regardless of its size.

The performance difference between prompting strategies, denoted as *Δ*, was defined as the absolute difference in micro-F1 scores.

Additionally, a category-specific error analysis was conducted to investigate failure patterns depending on the recommended miTNM staging. All analyses were performed using Python (NumPy 1.26.4, pandas 2.2.0, scikit-learn 1.4.0, statsmodels 0.14.1, matplotlib 3.8.2, seaborn 0.13.2) [17–21]. We estimated the 95% CI via bootstrap resampling with 10,000 iterations.

## Results

**Fig. 1:**
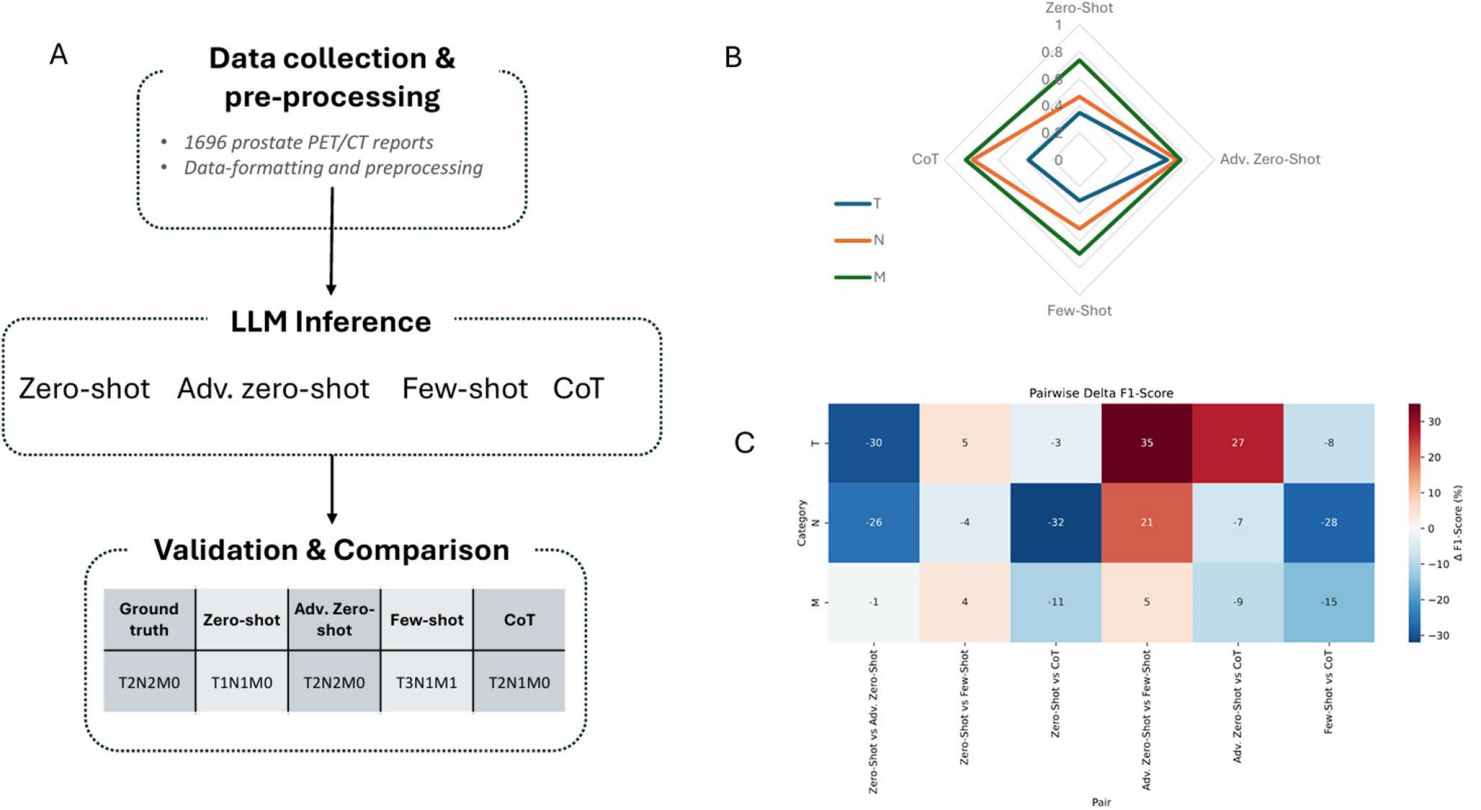
(**A**) Schematic overview of the end-to-end workflow for TNM classification of PET/CT reports. The process begins with report acquisition (*n* = 1696) and data preparation, followed by Zero-shot, adv. Zero-shot, few-shot and CoT prompting to predict T, N and M. Each prediction is then validated against expert-annotated ground truth labels. (**B**) Radar chart displaying the micro F1 scores for T, N and M. (**C**) Heatmap illustrating pairwise performance differences (in percentage points) among the prompting strategies for T, N and M. Positive (red) values indicate higher performance of the row model compared to the column model, while negative (blue) values indicate lower performance. This side-by-side comparison highlights the relative strengths and weaknesses of each prompting strategy, offering a straightforward way to identify which model performed best on individual staging components

The final analysis included 1,696 PSMA-PET/CT reports. The cohort’s clinical characteristics, including age (mean 72.7 ± 8.9 years), PSA levels (median 28.9 ng/ml), and the distribution of Gleason Scores and miTNM stages, are summarized in Table 1. The systematic evaluation focused on comparing four prompting strategies—Zero-shot, advanced Zero-shot, Few-shot, and Chain-of-Thought (CoT)—across the T, N, and M categories (Fig. 1).

**Table 1:** Distribution of age, PSA, Gleason Score, T, N and M stage among the study population

Category	Metric	Value	Percentage (%)
Age (Years)	Mean (SD)	72.7 (8.9)	
	Median [IQR]	73.0 [66.0–80.0]	
PSA (ng/ml)	Mean	236.7	
	Median [IQR]	28.9 [10.0-156.2]	
	**Stage/Score**	**Count**	
Gleason Score	< 7	96	5.7
	7	409	24.1
	8	425	25.1
	9–10	514	30.3
	Unknown	252	14.9
T (Tumor)	T2	891	52.5
	T3	596	35.1
	T4	209	12.3
N (Nodes)	N0	905	53.4
	N1	218	12.9
	N2	573	33.8
M (Metastasis)	M0	721	42.5
	M1	975	57.5

### T category

Adv. Zero-shot achieved the best micro-F1 (0.6480, 95% CI [0.6253, 0.6707]), outperforming Zero-shot (Δ = +0.3007), Few-shot (Δ = +0.3461), and CoT (Δ = +0.2706). CoT improved only slightly over Zero-shot (Δ = +0.0301). Macro metrics (precision/recall/F1) follow the same ranking: Advanced Zero-shot > CoT ≈ Zero-shot > Few-shot (Table 2).

**Table 2:** Classification performance for the T (Tumor) category across different prompting strategies

T	Zero-shot	adv. Zero-shot	Few-shot	CoT
Accuracy	0.3473 (95% CI: [0.3246, 0.3699])	0.6480 (95% CI: [0.6253, 0.6707])	0.3019 (95% CI: [0.2800, 0.3237])	0.3774 (95% CI: [0.3543, 0.4004])
Precision	0.1687 (95% CI: [0.1509, 0.1865])	0.6910 (95% CI: [0.6690, 0.7130])	0.3007 (95% CI: [0.2789, 0.3225])	0.3230 (95% CI: [0.3008, 0.3453])
Recall	0.2983 (95% CI: [0.2766, 0.3201])	0.5088 (95% CI: [0.4851, 0.5326])	0.1493 (95% CI: [0.1324, 0.1663])	0.3556 (95% CI: [0.3328, 0.3784])
macro F1	0.2060 (95% CI: [0.1867, 0.2252])	0.5157 (95% CI: [0.4919, 0.5395])	0.1522 (95% CI: [0.1352, 0.1693])	0.2727 (95% CI: [0.2515, 0.2939])
micro F1	0.3473 (95% CI: [0.3246, 0.3699])	0.6480 (95% CI: [0.6253, 0.6707])	0.3019 (95% CI: [0.2800, 0.3237])	0.3774 (95% CI: [0.3543, 0.4004])

The table compares Zero-shot, Advanced (adv.) Zero-shot, Few-shot, and Chain-of-Thought (CoT) approaches. Evaluation metrics include Accuracy, Precision, Recall, Macro F1, and Micro F1. All values are reported with their 95% confidence intervals, estimated via bootstrapping

### N category

CoT was superior with micro-F1 = 0.7895 (95% CI [0.7701, 0.8089]), yielding clear gains over Zero-shot (Δ = +0.3237) and a smaller but consistent gain over adv. Zero-shot (Δ = +0.0678). Few-shot offered only a modest improvement over Zero-shot (Δ = +0.0430). CoT also led on macro precision and recall (≈ 0.72–0.74) (Table 3).

**Table 3:** Classification performance for the N (Node) category across different prompting strategies

*N*	Zero-shot	adv. Zero-shot	Few-shot	CoT
Accuracy	0.4658 (95% CI: [0.4421, 0.4895])	0.7217 (95% CI: [0.7004, 0.7430])	0.5088 (95% CI: [0.4851, 0.5326])	0.7895 (95% CI: [0.7701, 0.8089])
Precision	0.3919 (95% CI: [0.3686, 0.4151])	0.7113 (95% CI: [0.6897, 0.7329])	0.4023 (95% CI: [0.3789, 0.4256])	0.7185 (95% CI: [0.6971, 0.7399])
Recall	0.5417 (95% CI: [0.5180, 0.5654])	0.7226 (95% CI: [0.7013, 0.7439])	0.4477 (95% CI: [0.4241, 0.4714])	0.7444 (95% CI: [0.7237, 0.7652])
macro F1	0.3652 (95% CI: [0.3423, 0.3881])	0.6612 (95% CI: [0.6387, 0.6837])	0.3715 (95% CI: [0.3485, 0.3945])	0.7186 (95% CI: [0.6972, 0.7400])
micro F1	0.4658 (95% CI: [0.4421, 0.4895])	0.7217 (95% CI: [0.7004, 0.7430])	0.5088 (95% CI: [0.4851, 0.5326])	0.7895 (95% CI: [0.7701, 0.8089])

Results are shown for Zero-shot, Advanced (adv.) Zero-shot, Few-shot, and Chain-of-Thought (CoT) prompting. The evaluation covers Accuracy, Precision, Recall, Macro F1, and Micro F1. All performance values include 95% confidence intervals estimated via bootstrapping

### M category

CoT again performed best with micro-F1 = 0.8420 (95% CI [0.8246, 0.8593]), exceeding adv. Zero-shot (Δ = +0.0938), Zero-shot (Δ = +0.1067), and Few-shot (Δ = +0.1468). Few-shot trailed both Zero-shot (Δ = −0.040) and advanced Zero-shot (Δ = −0.053). Macro precision/recall likewise favored CoT (≈ 0.86) (Table 4).

**Table 4:** Classification performance for the M (Metastasis) category across different prompting strategies

M	Zero-shot	adv. Zero-shot	few-shot	CoT
Accuracy	0.7353 (95% CI: [0.7143, 0.7563])	0.7482 (95% CI: [0.7276, 0.7689])	0.6952 (95% CI: [0.6733, 0.7171])	0.8420 (95% CI: [0.8246, 0.8593])
Precision	0.8023 (95% CI: [0.7834, 0.8213])	0.8071 (95% CI: [0.7883, 0.8259])	0.6884 (95% CI: [0.6664, 0.7105])	0.8591 (95% CI: [0.8426, 0.8757])
Recall	0.7685 (95% CI: [0.7484, 0.7886])	0.7794 (95% CI: [0.7597, 0.7991])	0.6892 (95% CI: [0.6671, 0.7112])	0.8604 (95% CI: [0.8439, 0.8769])
macro F1	0.7322 (95% CI: [0.7111, 0.7533])	0.7461 (95% CI: [0.7254, 0.7668])	0.6888 (95% CI: [0.6667, 0.7108])	0.8420 (95% CI: [0.8246, 0.8593])
micro F1	0.7353 (95% CI: [0.7143, 0.7563])	0.7482 (95% CI: [0.7276, 0.7689])	0.6952 (95% CI: [0.6733, 0.7171])	0.8420 (95% CI: [0.8246, 0.8593])

This table displays the results for Zero-shot, Advanced (adv.) Zero-shot, Few-shot, and Chain-of-Thought (CoT) strategies. Reported metrics are Accuracy, Precision, Recall, Macro F1, and Micro F1, presented together with 95% confidence intervals estimated via bootstrapping

Overall, best-in-class strategies by label group were adv. Zero-shot for T, and CoT for N and M. The largest pairwise improvements in micro-F1 were Zero-shot→CoT in N (+ 0.324) and Few-shot→CoT in M (+ 0.147). Across tasks, the 95% CIs of the top method per category show limited overlap with those of weaker baselines, indicating consistent advantages.

### Error analysis

**Fig. 2:**
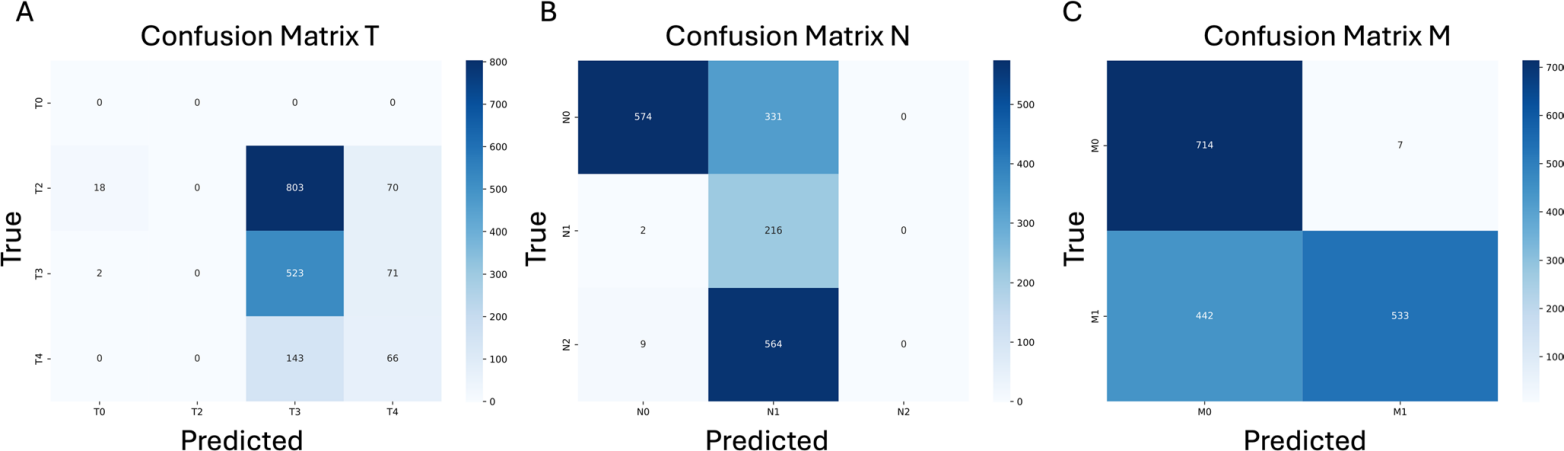
Confusion matrices for Zero-shot predictions for (**A**) T, (**B**) N and (**C**) M stage

Zero-shot prompting predominantly collapses predictions into the most frequent/central labels, yielding a strong T3 bias for T (many T2→T3 and T4→T3 errors), a collapse to N1 with no N2 predictions for N, and high specificity but poor sensitivity for M1 in M (Fig. 2).

**Fig. 3:**
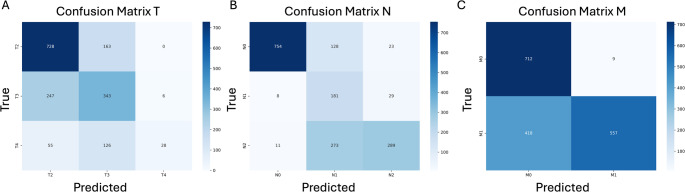
Confusion matrices for adv. Zero-shot predictions for (**A**) T, (**B**) N and (**C**) M stage

Advanced Zero-shot prompting predominantly produces diagonally dominant confusion matrices with most residual errors between adjacent classes. For T, correct calls dominate for T2/T3/T4. Errors are mainly T2→T3 and T4→T3; virtually no long-range T2↔T4 swaps. T4 remains under-called relative to T3. For N, class separation improves with clear recovery of N2 (N2→N2 = 289), though N2 is still often collapsed into N1 (N2→N1 = 273). For M, specificity is very high, while M1 sensitivity improves but is incomplete, indicating residual under-calling of metastasis (Fig. 3).

**Fig. 4:**
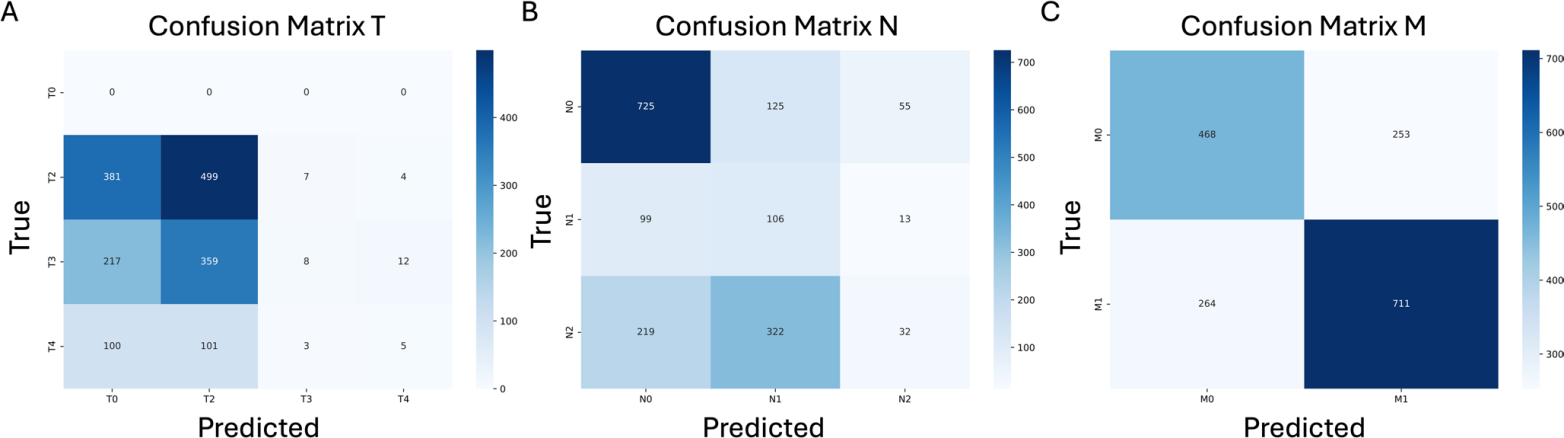
Confusion matrices for few-shot predictions for (**A**) T, (**B**) N and (**C**) M stage

Few-shot prompting predominantly yields only weakly diagonal confusion matrices, with substantial drift to adjacent classes and some residual long-range errors. T: Correct calls for T2/T3/T4 are modest, with strong symmetric confusion between T2→T3 and T3→T2. T4 is markedly under-called toward T2/T3, while the reverse long-range error is rare (T2→T4 = 4). N: Class separation is poor, and N2 rarely persists (N2→N2 = 32), collapsing mainly into N1 (322) and N0 (219); N1 itself is unstable (e.g., N1→N0 = 99). M: Errors are balanced, with comparable false positives and false negatives (M0→M1 = 253, M1→M0 = 264), indicating moderate specificity and incomplete sensitivity for metastasis detection (Fig. 4).

**Fig. 5:**
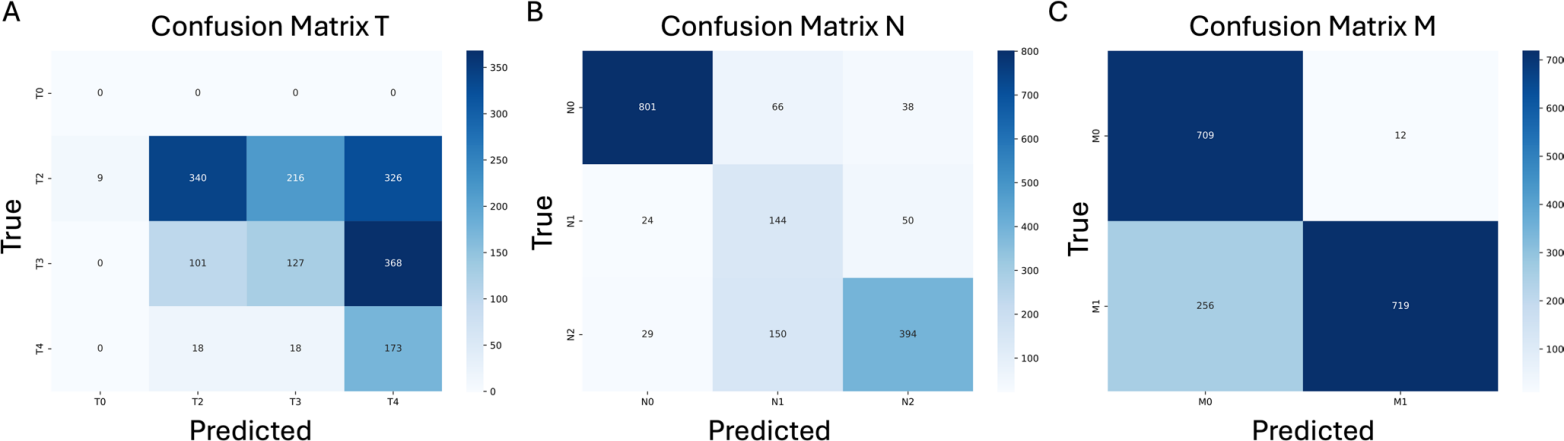
Confusion matrices for CoT predictions for (**A**) T, (**B**) N and (**C**) M stage

Chain-of-thought prompting predominantly yields diagonally dominant confusion matrices, with the clearest gains for N and M, and a residual upward bias in T staging. In the T matrix, correct calls increase but many true T2 and T3 cases are upgraded to T4 (T2→T4 = 326; T3→T4 = 308), while downgrades from T4 are comparatively rare (T4→T2 = 18; T4→T3 = 18). Long-range off-diagonal errors outside adjacent classes are minimal. For nodal status, separation is strong: N0 specificity is high (N0→N0 = 801) and N2 is well recovered (N2→N2 = 394), with the remaining errors mostly adjacent collapses (N2→N1 = 150; N0→N1 = 66). For metastasis, specificity is excellent (M0→M0 = 709 vs. M0→M1 = 12) and M1 sensitivity is improved yet still imperfect, reflected by residual under-calling (M1→M0 = 256). Overall, CoT delivers the most consistent improvements for N and M, while T staging remains biased toward predicting the higher T4 category (Fig. 5).

## Discussion

In this retrospective analysis, we investigated the use of large language models (LLMs) for TNM staging of prostate cancer from PSMA PET/CT reports, with particular focus on their consistency with the PROMISE v2 framework. To our knowledge, this represents one of the first systematic explorations of LLMs in molecular imaging–driven oncologic staging of prostate cancer. By leveraging structured criteria such as PROMISE v2, LLMs may potentially contribute to standardizing interpretation and providing reproducible staging outputs, although further multi-institutional studies are required to confirm their impact on mitigating inter-reader variability [10].

Our results reveal that while LLMs excel at identifying nodal (N) and metastatic (M) involvement, local T-category staging remains highly challenging. This performance gap is likely rooted in the linguistic complexity of radiology reports. While M-staging often relies on clear, affirmative statements (e.g., “bone metastasis in the pelvis”), T-staging requires the interpretation of subtle anatomical nuances and ‘hedged’ language (e.g., “focal suspicion of minimal extracapsular extension”). For an 8B-parameter model, these semantic subtleties between miT2 and miT3a are difficult to distinguish, whereas the more binary nature of N/M-staging favors the Chain-of-Thought approach.

We evaluated four prompting strategies across T, N, and M staging: Zero shot, advanced Zero shot, few shot, and chain of thought. Local tumor classification proved the most challenging task. The Zero-shot approach achieved a limited accuracy of 0.35, whereas both few-shot and chain-of-thought prompting strategies resulted in even lower performance. Specifically, even providing five clinically representative examples was insufficient to capture the high linguistic variability of the reports. The model tended to over-index on the specific patterns of these examples rather than abstracting the underlying PROMISE v2 rules. Nodal staging showed the greatest benefit from prompting, as chain of thought prompting surpassed Zero shot prompting with an accuracy of 0.79 compared to 0.47. Metastatic staging yielded consistently high performance across all strategies, with chain of thought prompting achieving the highest accuracy of 0.84 and precision of 0.86. Overall, these findings indicate that the most effective prompting strategy varies by staging category, with chain of thought prompting being particularly advantageous for nodal and metastatic classification. Our qualitative analysis revealed that discrepancies in the T-category frequently involved misinterpretations of specific terminology. For example, the model overestimated a miT2 case as miT3, likely misconstruing “multifocal prostate cancer” as evidence of extracapsular extension. Conversely, underestimation occurred in a miT4 case where the report stated “infiltration of the external iliac vein not excluded”; the model predicted miT3a, failing to recognize this potential infiltration as a T4 criterion. Furthermore, a case of “prostate cancer” with “suspicion of seminal vesicle infiltration” (miT3) was incorrectly classified as miT0, demonstrating a failure to correctly extract the primary diagnosis from the text.

Regarding lymph node (N) staging, errors ranged from missed extractions to hallucinations. In one severe false-negative instance (miN2 misclassified as miN0), the model ignored a detailed list of “multiple, PSMA-expressing lymph node metastases” in bilateral iliac regions. Conversely, the model produced a false-positive miN1 prediction for a miN0 case where the text explicitly stated “none” for both pelvic and extrapelvic nodes. In the M-category, errors were often driven by terminology mapping, such as failing to recognize anatomical abbreviations (e.g., “LWK” for lumbar vertebrae or “BWK” for thoracic vertebrae) as skeletal sites (miM1). Notably, the model occasionally violated specific PROMISE v2 rules by misclassifying PSMA-negative osteosclerotic lesions as miM1, highlighting that only PSMA-positive lesions should define the miTNM stage. Previous research in oncologic imaging has predominantly concentrated on deep learning approaches applied directly to image data, aiming at lesion detection and quantitative analysis [22, 23]. In contrast, our study emphasizes the potential of text-based AI models to interpret narrative PET/CT reports and convert them into structured TNM classifications. Comparable strategies have already shown promise in breast cancer or lung cancer staging [14, 16], and our findings extend this application to prostate cancer. Importantly, the use of LLMs to extract PROMISE-based staging features from narrative PET/CT reports may enhance reproducibility by standardizing the reporting structure, even though the underlying interpretation remains dependent on the original clinical report. The prognostic relevance of PROMISE v2 parameters has been well established, with studies demonstrating associations between miTNM categories, metastatic distribution, and oncologic outcomes [10, 24, 25].

The clinical utility of our approach lies in two primary scenarios: First, as a real-time decision support tool during report drafting, where the LLM can provide a “second look” to ensure miTNM staging is explicitly and correctly assigned. Second, for large-scale retrospective research, where manual staging of thousands of reports is unfeasible. By automating this process, LLMs can significantly reduce inter-physician variability and improve the reproducibility of clinical registries.

If LLMs can consistently capture and classify these features from PET/CT reports, they may serve as a scalable tool for outcome prediction and patient stratification. Moreover, structured extraction of staging data by LLMs could enable large-scale retrospective analyses of outcomes in prostate cancer, further validating PROMISE v2 in real-world populations. Nonetheless, several challenges remain. First, the accuracy of LLM-based staging depends on the quality and consistency of PET/CT reporting, which can vary across institutions [26–29]. Second, while LLMs are capable of applying structured frameworks such as PROMISE v2, their performance in handling ambiguous or incomplete reports requires careful evaluation. Finally, prospective validation in clinical trials will be required before this approach can be recommended for routine clinical use.

Several limitations must be acknowledged. First, this is a single-center study, and the model’s performance might differ at institutions with different dictation styles. Second, our “ground truth” was derived from the reports themselves. While this evaluates the LLM’s ability to extract information (NLP performance), it does not replace clinical validation against histopathology. Future studies should focus on external validation and the integration of pathological outcomes.

In summary, our findings demonstrate the feasibility of using LLMs for automated TNM staging of prostate cancer from PSMA PET/CT reports. By integrating PROMISE v2 into LLM workflows, these models could standardize reporting, enhance reproducibility, and support large-scale clinical research workflows. Future studies should focus on prospective validation, assessment of prognostic value, and evaluation of integration into clinical decision support systems.

## Conclusion

This study demonstrates the feasibility of applying large language models for automated prostate cancer staging from PSMA-PET/CT reports within the PROMISE v2 framework. Among the evaluated prompting strategies, advanced Zero-shot prompting proved most effective for tumor classification, while chain-of-thought prompting yielded superior results for nodal and metastatic staging. These findings highlight that LLM performance is highly context-dependent, with specific prompting approaches offering clear advantages for individual staging categories.

LLMs can standardize prostate cancer staging by improving reproducibility and enabling broader data analyses. Yet, variability in report quality and the need for prospective validation remain key hurdles.

In conclusion, integrating PROMISE v2 into LLM workflows offers a scalable path toward consistent and clinically meaningful staging, with potential to advance automated data processing in oncologic research.

## Supplementary Information

Below is the link to the electronic supplementary material.

Supplementary Material 1.

Supplementary Material 2.

## Data Availability

The datasets generated and analyzed during the current study are not publicly available due to institutional data protection regulations and patient privacy considerations but are available from the corresponding author upon reasonable request and subject to approval by the relevant institutional authorities.
