# Supplementary material for: LLM-powered prostate cancer staging from PSMA-PET/CT reports using PROMISE v2
Source: Eur J Nucl Med Mol Imaging. 2026 Mar 21;53(8):5050–9. doi: 10.1007/s00259-026-07847-w (PMC13249623; doi:10.1007/s00259-026-07847-w)
Supplement: Supplementary file 2 — Supplementary Material 2. [file 259_2026_7847_MOESM2_ESM.docx]

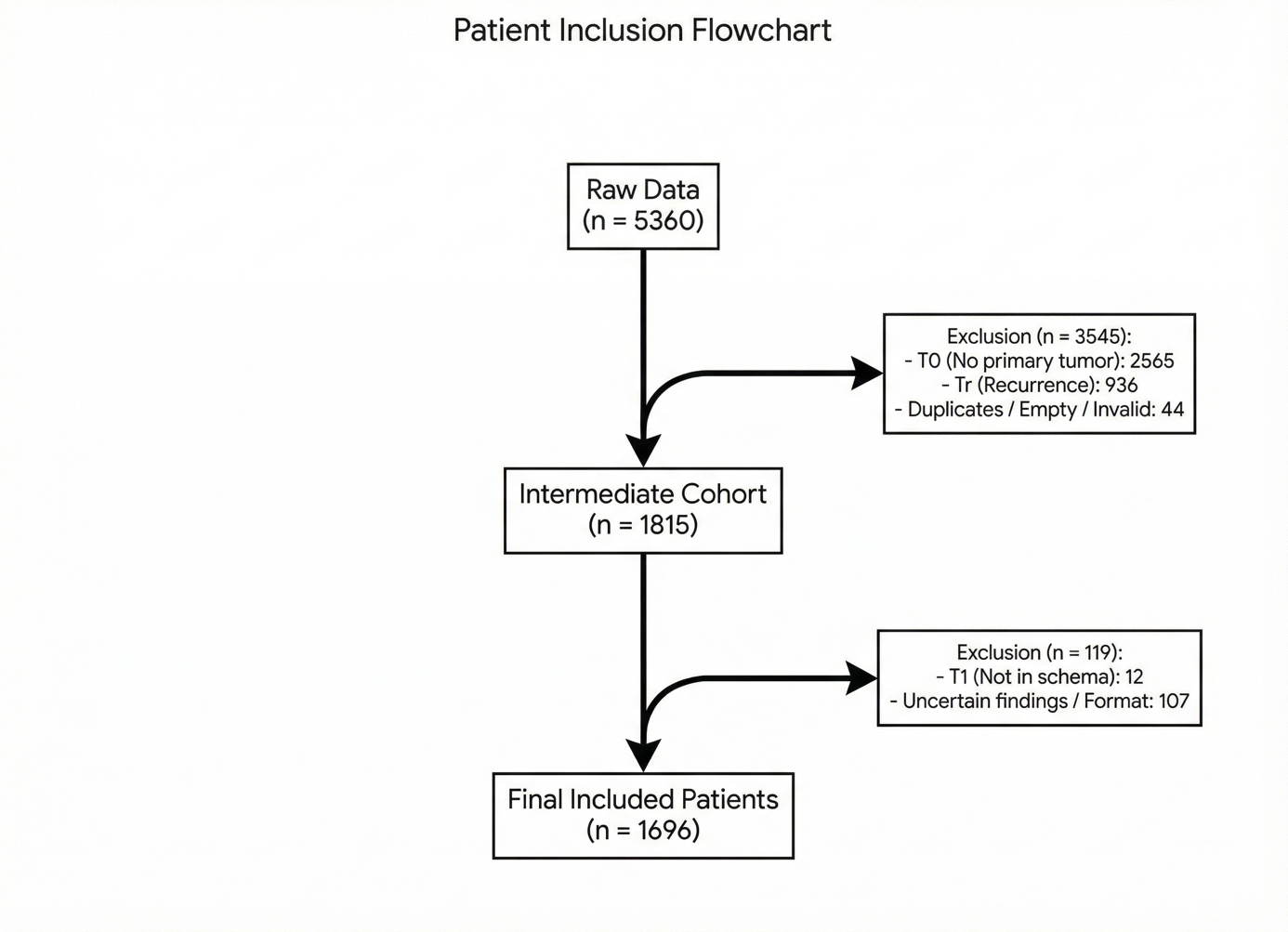


*Suppl. Figure 1: Study flowchart illustrating the patient selection process. Out of 5,360 initial entries, a total of 3,664 cases were excluded based on predefined criteria (e.g., recurrence, lack of primary tumor, or ambiguous reports), resulting in a final cohort of 1,696 patients included in the analysis*
